# Inhibitory Effects of Acetylmelodorinol, Chrysin and Polycarpol from *Mitrella kentii* on Prostaglandin E_2_ and Thromboxane B_2_ Production and Platelet Activating Factor Receptor Binding

**DOI:** 10.3390/molecules17054824

**Published:** 2012-04-26

**Authors:** Sakina Saadawi, Juriyati Jalil, Malina Jasamai, Ibrahim Jantan

**Affiliations:** Drug and Herbal Research Centre, Faculty of Pharmacy, Universiti Kebangsaan Malaysia, Jalan Raja Muda Abdul Aziz, 50300 Kuala Lumpur, Malaysia

**Keywords:** *Mitrella kentii*, chrysin, acetylmelodorinol, polycarpol, prostaglandin E_2_ (PGE_2_), thromboxane B_2_ (TXB_2_), platelet activating factor (PAF) antagonist

## Abstract

Acetylmelodorinol, chrysin and polycarpol, together with benzoic acid, benzoquinone and stigmasterol were isolated from the leaves of *Mitrella kentii* (Bl.) Miq. The compounds were evaluated for their ability to inhibit prostaglandin E_2_ (PGE_2_) and thromboxane B_2_ (TXB_2_) production in human whole blood using a radioimmunoassay technique. Their inhibitory effect on platelet activating factor (PAF) receptor binding to rabbit platelet was determined using ^3^H-PAF as a ligand. Among the compounds tested, chrysin showed a strong dose-dependent inhibitory activity on PGE_2_ production (IC_50_ value of 25.5 µM), which might be due to direct inhibition of cyclooxygenase-2 (COX-2) enzymatic activity. Polycarpol, acetylmelodorinol and stigmasterol exhibited significant and concentration-dependent inhibitory effects on TXB_2_ production with IC_50_ values of 15.6, 19.1 and 19.4 µM, respectively, suggesting that they strongly inhibited COX-1 activity. Polycarpol and acetylmelodorinol showed strong dose-dependent inhibitory effects on PAF receptor binding with IC_50_ values of 24.3 and 24.5 µM, respectively.

## 1. Introduction

The hydrolysis of the sn-2 position of membrane glycerophospholipids to liberate arachidonic acid (AA), a precursor of eicosanoids including prostaglandins (PGs) and leukotrienes (LTs), is catalyzed by phospholipase A_2_ (PLA_2_). Prostaglandin E_2_ (PGE_2_), a metabolite of AA through the cyclooxygenase-2 (COX-2) pathway, has received great attention because of its role and contribution to inflammation. It is also involved in a variety of other functions, such as vasodilation, febrile responses, and altered microvascular permeability [1]. Thromboxane A_2_ (TXA_2_) is an oxidation product derived from AA in cyclooxygenase and thromboxane synthase dependent reactions. TXA_2_ is rather unstable and is rapidly hydrolyzed into the almost inactive, stable and more measurable metabolite thromboxane B_2_ (TXB_2_) [2]. The determination of serum TXB_2_ production by platelets following blood coagulation is a specific and most common method for evaluation of COX-1 activity in human and other species [3]. TXA_2_ is a vasoconstrictor and a promoter of platelet aggregation and plays an important role in the maintenance of vascular homeostasis. TXA_2_ has been postulated to be a mediator contributing to the pathophysiology of several of disease processes such as thrombosis, atheroscleoris and myocardial ischemia [4]. The radioimmunoassay technique (RIA) is the most common way to quantify PGE_2_ and TXB_2_ in plasma as it is able to detect and quantify the antigen-antibody interaction [5].

PAF is a potent glycerophospholipid mediator, involved in several pathophysiological conditions such as inflammation [6], allergy [7], asthma [8] and thrombosis [9]. Specific receptors for PAF have been reported in a variety of cell membranes including those from platelets [10]. Therefore, compounds which inhibit the specific binding between PAF and receptors may be used as leads in the development of therapeutic agents in a variety of inflammation, respiratory, immunological and cardiovascular disorders [11]. It has been reported that the synthesis of both PAF and prostanoids shares common metabolic pathways, but the relationships between them have not been defined extensively [12]. Some authors have suggested that TXA_2_ can be the mediator of the PAF effects in some pathophysiologic conditions [13]. In addition, PAF induce an increased PGE_2_ synthesis in cultured rat mesangial cells. Moreover, they suggested an intermediate PAF role in the prostanoids secondary production [14].

*Mitrella kentii* (Bl.) Miq. is a tree-climbing liana belonging to the custard apple family called Annonaceae. Its synonyms are *Melodorum pisocarpum* and *Me. elegans*, while the common (Indonesian) name is ‘kiawi’. The plant is found in the tropics, especially in the Asia-Pacific regions. It is used traditionally in Malaysia as a drink in the form of a root decoction to treat fever [15]. Earlier phytochemical investigation on the bark of the plant had reported the isolation of four alkaloids, namely liriodenine, anonaine, and asimilobinem and aequaline [16]. Terpenylated dihydrochalcones: (−)-neolinderatin, (−)-linderatin and 2',6'-dihydroxy-4' methoxydihydrochalcone, and (+)-catechin have also been identified in the stem bark of *M. kentii* [17]. 

In our screening study to identify compounds from tropical plants as potential anti-inflammatory agents, we observed that the methanol extract of the leaves of *M. kentii* showed strong inhibitory effects on PGE_2_ and TXB_2_ production in human whole blood (>50.0% inhibition) and PAF receptor binding to rabbit platelets (>60% inhibition). In this paper, we report on the isolation of acetylmelodorinol, chrysin and polycarpol, together with benzoic acid, benzoquinone and stigmasterol, from this plant and their ability to inhibit COX-1 and COX-2 activities through inhibition of the production of TXB_2_ and PGE_2_ in human whole blood and displacement of ^3^H-PAF-specific binding in washed rabbit platelets.

## 2. Results and Discussion

### 2.1. Isolation and Identification of Compounds

**Figure 1:**
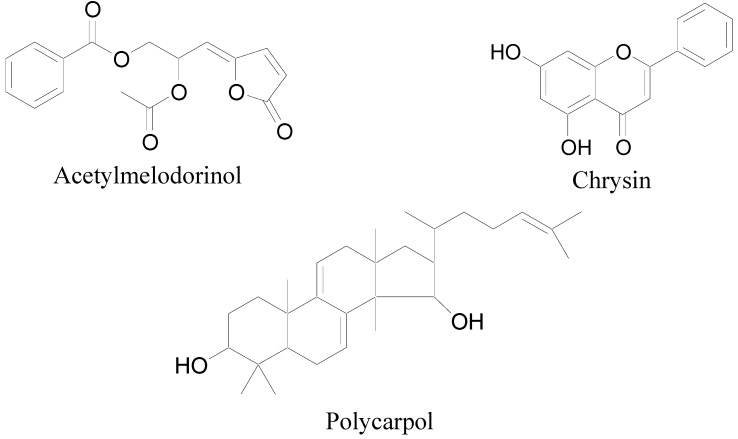
Chemical structures of acetylmelodorinol, chrysin and polycarpol.

In this study, six compounds have been isolated from the leaves of *Mitrella kentii* by chromatographic techniques. The compounds were identified as benzoic acid, acetylmelodorinol [18], chrysin [19], benzoquinone [20], stigmasterol [21] and polycarpol [18] by comparing their physicochemical and spectroscopic properties with literature values, in addition to their 2D NMR spectra and ESI-MS spectra (See Figure 1 for the structures of acetylmelodorinol, chrysin and polycarpol). Alkaloids and terpenylated dihydrochalcones reportedly identified in the bark of the plant in previous studies were not found in the leaves of the plant [16,17].

### 2.2. Inhibition of Production of PGE_2_ and TXB_2_

The cell viability test carried out to evaluate the cytotoxicity of the compounds on the blood cells at 1.25 and 10.0 µg/mL indicated that the blood cells were viable (>95%) after 24 h incubation. The compounds were investigated for their inhibitory effects on production of PGE_2_ and TXB_2_ in whole blood at 10 µg/mL. Compounds which showed strong inhibitory activity (>50% inhibition) were subsequently tested at serial concentrations for determination of their IC_50_ values. Amongst the compounds tested, chrysin exhibited the strongest inhibitory effect on the production of PGE_2_ (63.6% inhibition) induced by lipopolysaccharide (LPS) in whole blood (Table 1). Chrysin was further investigated at various concentrations and it showed dose-dependent response, *i.e.*, as the concentration of the compound increased, the percentage inhibition increased (Table 2). However, the IC_50_ value of chrysin (25.5 µM) was higher than that of indomethacin (9.5 µM), a potent cyclooxygenase inhibitor [22]. The inhibition of PGE_2_ production might be due to direct inhibition of cyclooxygenase-2 (COX-2) enzymatic activity, possibly by the anti-oxidant effects of the compound or related to its capacity to bind COX-2 active site. However, phospholipase A_2_ (PLA_2_) may also be as a potential target for the compound. 

**Table 1:** Percentageinhibition (%) of compounds isolated from *Mitrella kentii* at 10 µg/mL on PGE_2_ production in human whole blood induced by LPS.

Compound	% Inhibition
Benzoic acid	41.5 ± 7.4 *
Acetylmelodorinol	23.1 ± 6.0 *
Chrysin	63.6 ± 6.0 *
Benzoquinone	29.9 ± 14.3 *
Stigmasterol	36.4 ± 5.6 *
Polycarpol	34.7 ± 10.9 *
Indomethacin	83.8 ± 4.9 *

Values are presented as mean ± SD (n = 3); * *P* < 0.05 as compared with control.

**Table 2:** Percentage inhibition (%) and IC_50_ values of chrysin from *Mitrella kentii* on PGE_2_ production in human whole blood induced by LPS.

Compounds	Concentrations (µg/mL)				IC_50_ µg/mL (µM)
	10	5	2.5	1.25	
Chrysin	63.6	38	14.2	5.8	6.5 ± 0.3 (25.5)
Indomethacin	83.8	63.1	32.4	12.35	3.4 ± 0.5 (9.5)

Values are presented as mean ± SD (n = 3); IC_50_ values in µM are presented in parentheses.

In the TXB_2_ assay, acetylmelodorinol displayed the highest percentage inhibition (61.5%), followed by polycarpol (60.6%), stigmasterol (54.3%) and crysin (51.2%) (Table 3). The percentage inhibition of the compounds at different concentrations is shown in Table 4. The results showed that the compounds inhibited TXB_2 _production in human whole blood in a dose-dependent manner, *i.e.*, as the concentrations of the compounds increased the percentages of inhibition increased (Table 4). The IC_50_ values for polycarpol, acetylmelodorinol, stigmasterol and crysin were 15.6, 19.2, 19.4 and 39.3 µM, respectively. The IC_50_ value of polycarpol was comparable to that of indomethacin (12.8 µM). Since the measurement of serum TXB_2_ production by the platelets following blood coagulation is a specific test for assessment of COX-1 activity, the results suggest that the inhibitory effect of the compounds could be due to direct inhibition of COX-1 enzymatic activity.

Chrysin, a flavone devoid of carboxylic acid funtional group, which showed strong inhibitory effects on PGE_2_ and TXB_2_ production in human whole blood could be used as a lead compound in the development of COX inhibitors. Chrysin has been reported to have several biological activities including antioxidant, anti-allergic, anti-inflammatory, anticancer, antiestrogenic, hypoglycemic and anxiolytic activities [23]. Of all the compounds tested, polycarpol showed the strongest inhibitory activity on TXB_2_ production, reflecting its specific inhibition of COX-1 activity. The butenolide, acetylmelodorinol, which was the major compound in *M. kentii*, also showed significant inhibitory activity on TXB_2_ production in human whole blood. Stigmasterol which showed moderate effect on TXB_2_ production has been reported to possess strong anti-inflammatory activity [24]. In a study conducted by Gomez *et al.* [25], stigmasterol was found to be more effective as topical anti-inflammatory agent in acute than in chronic processes.

**Table 3:** Percentageinhibition (%) of compounds from *Mitrella kentii* at 10 µg/mL on TXB_2_ production in human whole blood.

Compound	% Inhibition
Benzoic acid	42.1 ± 6.4 *
Acetylmelodorinol	61.5 ± 7.0 *
Chrysin	51.2 ± 4.0 *
Benzoquinone	33.1 ± 10.6 *
Stigmasterol	54.3 ± 10.4 *
Polycarpol	60.6 ± 7.8 *
Indomethacin	73.9 ± 6.5 *

Values are presented as mean ± SD (n = 3); * *P* < 0.05 as compared with control.

**Table 4:** Percentage inhibition (%) and IC_50_ values of compounds isolated from *Mitrella kentii* on TXB_2_ production in human whole blood.

Compound	Concentrations (µg/mL)				IC_50_ µg/mL (µM)
	10	5	2.5	1.25	
Acetylmelodorinol	61.5	47.3	18.5	7.4	5.8 ± 0.1 (19.2)
Stigmasterol	54.3	30.2	10.4	5.8	8.0 ± 1.1 (19.4)
Polycarpol	60.6	30.8	18.3	3.2	6.9 ± 0.6 (15.6)
Chrysin	51.2	23	16.7	7.5	10.1 ± 0.8 (39.3)
Indomethacin	73.9	50.7	29.2	10.5	4.6 ± 0.1 (12.8)

Values are presented as mean ± SD (n = 3); IC_50_ values in µM are presented in parentheses.

### 2.3. Effect of the Compounds on 3H-PAF Receptor Binding

**Figure 2:**
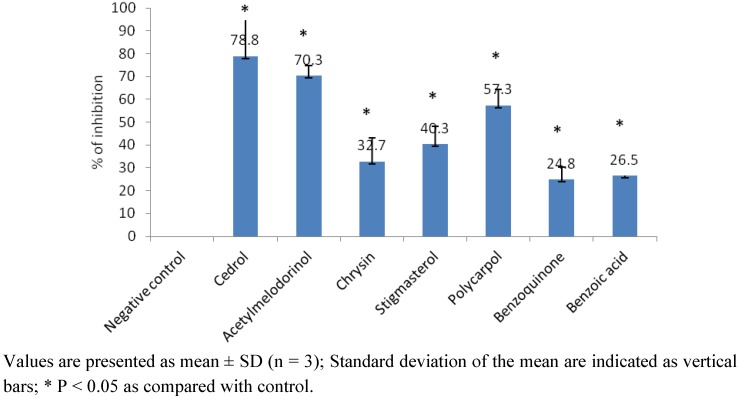
Percentage inhibition (%) of the compounds isolated from *Mitrella kentii* on PAF receptor binding to rabbit platelets at 18.2 µg/mL.

**Figure 3:**
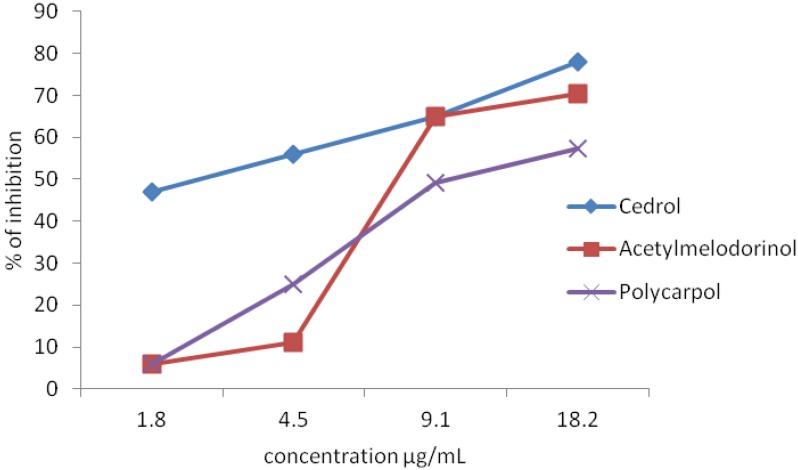
Inhibitory effects of acetylmelodorinol, polycarpol and cedrol on PAF receptor binding to rabbit platelets at different concentrations.

The inhibitory effect of the compounds on PAF receptor binding to rabbit platelets at the concentration of 18.2 µg/mL is shown in Figure 2. Among the compounds studied, acetylmelodorinol and polycarpol showed significant inhibitory effects on the PAF receptor binding with percentages of inhibition of 70.3 and 57.3%, respectively. The percentage inhibition of acetylmelodorinol was comparable to that of the positive control, cedrol (78.8%), a known PAF antagonist from natural sources [26]. The inhibitory effects of the compounds were then evaluated at various concentrations and the IC_50_ values of acetylmelodorinol and polycarpol were determined by probit analysis as 24.5 and 24.3 µM, respectively (Table 5). Acetylmelodorinol and polycarpol showed relatively strong PAF antagonistic activity and their dose-dependent responses are shown in Figure 3. The compounds inhibited the specific binding between PAF and receptors and thus they may be used as leads in the development of therapeutic agents in a variety of inflammation, respiratory, immunological and cardiovascular disorders [27]. Polycarpol has been shown to exhibit interesting antitrypanosomal activity but its anti-inflammatory effect has not been reported [28]. Acetylmelodorinol has been reported to exhibit general antiproliferative activity toward tumor cells [18].

**Table 5:** IC_50_ values of the active compounds from *Mitrella kentii* on PAF receptor binding to rabbit platelets.

Compound	IC_50_ µg/mL (µM)
Acetylmelodorinol	7.4 ± 0.4 (24.5)
Polycarpol	10.7 ± 0.3 (24.3)
Cedrol	2.9 ± 0.5 (13.0)

Values are presented as mean ± SD (n = 3). IC_50_ values in µM are presented in parentheses.

## 3. Experimental

### 3.1. General

Hexane, ethyl acetate and methanol used were analytical grades. Radiolabelled PGE_2_ ([3H]-PGE_2_, 50 µCi mmol^−1^) and TXB_2_ ([3H]-TXB_2_, 25 µCi mmol^−1^) were purchased from Amersham (Buckinghamshire, UK). Unlabelled PGE_2_, unlabelled TXB_2_, anti-PGE_2 _and anti-TXB_2_ were obtained from Sigma Chemical Co. (St. Louis, MO, USA). Radiolabeled PAF (1-*O*-alkyl-2-acetyl-*sn*-glycero-3-phosphocholine, 125 Ci/mmol) was purchased from Amersham. Unlabeled PAF and cedrol were obtained from Sigma Chemical Co. Bovine Serum Albumin (BSA) was purchased from Boehringer Mannheim Co. (Mannheim, West Germany). Other chemicals were purchased from Merck Co. (Darmstadt, Germany) and BDH Laboratory Supplies (Poole, UK). Ethylenediamine tetraacetic acid (EDTA) 2% was used as anticoagulant. Lipopolysaccharide (LPS) 1 mg/mL was used as prostaglandin endoperoxide synthesis induction in whole blood. Phosphate buffer solutions (PBS) 0.01 M, pH 7.4 was used for assays buffer. Dextran charcoal (0.4% dextran, 2% charcoal) was used to separate the free and bound ligand. Scintillation cocktail was made up of 2,5-diphenyloxazole (PPO, 0.26%), 2,2'-*p*-phenylene-bis(5-phenyloxazole (POPOP, 0.006%), toluene (500 mL) and Triton X (250 mL). Radioactivity was measured by a liquid scintillation counter (LSC) (Packard Tri-Carb, models 2100TR/2300TR, Hamburg, Germany). Melting points were determined using a XSP-12 model 500X hot stage melting point apparatus equipped with a microscope, and were uncorrected. Electrospray ionization mass spectrometry (ESI-MS) was performed on MicroTOF-Q mass spectrometer (Bruker, Coventry, UK). The ^1^H-NMR (400 MHz) and ^13^C-NMR (100 MHz) spectra were recorded on a JEOL NMR spectrometer (JNM-GX, Eching, Germany) in CDCl_3_ or CD_3_OD with TMS as internal standard.

### 3.2. Plant Material

Fresh leaves of *Mitrella kentii* were collected from the Angsi mountain forest in Negeri Sembilan, Malaysia in July 2008 and identified by Dr Kamarudin Mat Salleh, Faculty of Science and Technology, Universiti Kebangsaan Malaysia (UKM). A voucher specimen (AZ 69) was deposited at the Herbarium of Faculty of Science and Technology, UKM.

### 3.3. Extraction and Isolation of Compounds

Dried ground leaves of *M. kentii* (1,000 g) were extracted successively with hexane (3 × 2.5 L, 24 h each), ethyl acetate (3 × 2.5 L, 24 h each) and methanol (3 × 2.5 L, 24 h each) using maceration technique. The solvents were then evaporated using a rotatory evaporator to yield hexane (14.9 g, 1.5%), ethyl acetate (29.5 g, 3.0%) and methanol (67.8 g, 6.8%) extracts, respectively. The extracts were subjected to column chromatography (CC) on silica gel 60 (230–400 mesh), eluted with a gradient system of *n*-hexane, ethyl acetate and methanol to afford six known compounds. The hexane extract yielded stigmasterol (a white amorphous solid, 52.0 mg) and polycarpol (a white amorphous solid, 43.0 mg), the ethyl acetate extract yielded benzoic acid (6.1 mg), acetylmelodorinol (colorless needles, 1.0 g), chrysin (a light yellow powder, 500.0 mg) and benzoquinone (colorless crystals, 2.0 mg), while acetylmelodorinol (54.0 mg) and chrysin (35.2 mg) were isolated from the methanol extract. The compounds were identified by comparison of their physicochemical and spectroscopic properties with literature values. Purity of the compounds was >95%, based on their physicochemical properties, NMR and ESI-MS data.

### 3.4. Cell Viability

Cell viability was determined by the standard trypan blue exclusion method. The blood cells (1 × 10^6^/mL) were incubated with 1.25 and 10.0 µg/mL of compounds, each in triplicate at room temperature for 24 h. The blue dye uptake was an indication of cell death. The percentage viability was calculated from the total cell counts.

### 3.5. Radioimmunoassay for Prostaglandin E_2_ (PGE_2_) and Thromboxane B_2_ (TXB_2_)

Radioimmunoassay was carried out to determine the levels of PGE_2_ and TXB_2_ productions by blood cells following incubation with compounds and coagulation according to the modified method of Patrignani *et al.* [29]. The use of human blood was approved by the Ethics Committee of Universiti Kebangsaan Malaysia (UKM) (approval no. FF-168-2007). Radioimmunoassay procedures were carried out in triplicate for each compound. 

#### 3.5.1. Preparation of Standards

A series of concentrations of PGE_2_ and TXB_2_ standards were prepared, ranging from 2.45–240 and 2.05–500 pg/0.1 mL, respectively. One hundred µL of PGE_2_ standard solution was added to 100 µL of anti-PGE_2_ and 100 µL of [3H]- PGE_2_. Meanwhile, 100 µL of TXB_2 _standard solution was added to 100 µL of anti-TXB_2_ and 100 µL of [3H] - TXB_2_. The mixtures were incubated at 4 °C for 18–24 h. After incubation, the mixtures were added with 200 µL of dextran charcoal and were incubated again for 10 min. After centrifugation at 2000× g for 15 min at 4 °C, 3 mL of liquid scintillation cocktail was added to 300 µL of supernatant. The radioactivity was measured by a liquid scintillation counter. 

#### 3.5.2. Prostaglandin E_2_ Radioimmunoassay

Briefly, venous blood was obtained in polypropylene tube containing 10% (v/v) of 2% EDTA by aseptic vein puncture from healthy human volunteers who fulfilled the following inclusion criteria: non-smoker, fasted overnight and did not take any medicine or supplements within the last two weeks. One mL of blood was incubated at 37 °C for 24 h with 10 µL of LPS and 10 µL of serial dilutions of each compound in DMSO and ethanol (1:1 ratio) (1.25–10 µg/mL) or control. DMSO and ethanol (1:1 ratio) was used as a negative control and indomethacin, a known cyclooxygenase inhibitor was used as a positive control. After incubation, the blood was centrifuged at 2,000 × g for 10 min at 4 °C to separate the plasma. The reaction mixtures consisted of 100 µL of plasma, 100 µL of anti-PGE_2_ and 100 µL of [3H]-PGE_2 _were incubated at 4 °C for 18–24 h. After incubation, the mixtures were added with 200 µL of dextran charcoal and were incubated again for 10 min. The final concentrations of the samples in the mixture were 10.0, 5.0, 2.5 and 1.25 µg/mL. After centrifugation at 3,000 × g for 15 min at 4 °C, 3 mL of liquid scintillation cocktail was added to 300 µL of supernatant. The radioactivity was measured by a liquid scintillation counter. 

#### 3.5.3. Thromboxane B_2_ Radioimmunoassay

Thromboxane B_2_ assay was carried out similarly to the PGE_2_ assay. In this assay, 1 mL of blood mixed with 10 µL of serial dilutions of each compound in DMSO and ethanol (1:1 ratio) (1.25–10 µg/mL) or control was allowed to clot for 60 min at 37 °C. DMSO and ethanol (1:1 ratio) was used as a negative control and indomethacin, a known cyclooxygenase inhibitor was used as a positive control. The blood was centrifuged at 2,000 × g for 10 min at 4 °C to separate the serum as supernatant. The reaction mixtures consisted of 100 µL of serum, 100 µL of anti-TXB_2_ and 100 µL of [3H]-TXB_2_ were incubated at 4 °C for 18–24 h. After incubation, the mixtures were added with 200 µL of dextran charcoal and were incubated again for 10 min. The final concentrations of the samples in the mixture were 10.0, 5.0, 2.5 and 1.25 µg/mL. After centrifugation at 3,000 × g for 15 min at 4 °C, 3 mL of liquid scintillation cocktail was added to 300 µL of supernatant. The radioactivity was measured by liquid scintillation counter. 

#### 3.5.4. Calculation of PGE_2_ and TXB_2_ Concentrations

The readings obtained for each set of triplicate were averaged. The net counts for all standards and samples were calculated by substracting the value of the antibody binding to the antigen in the sample (Bx) with non specific binding (Nc). The normalized percentage bound (% B/Bo) for each standard and sample (Bx) were calculated as follows:


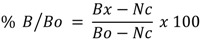


The % B/Bo for each standard *versus* the corresponding picogram (pg) concentration of PGE_2_ and TXB_2_ were plotted using semi-logarithmic graph. The concentrations of PGE_2_ and TXB_2_ in each sample were determined by interpolation from the standard curve. Percentage inhibition of samples was obtained as follows:


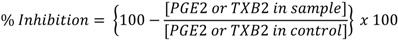


### 3.6. PAF Receptor Binding Inhibitory Assay (PAF Assay)

The assay was carried out according to the method as described previously by Jantan *et al.* [30]. The procedure was approved by the Animal Ethics Committee of UKM (approval no. FSKB/2007/Juriyati/10-July/192). The reaction mixtures consisted of 200 µL of washed rabbit platelet suspension, 25 µL of ^3^H-PAF (2.0nM) with or without unlabeled 25 µL of PAF (2.0 µM) and 25 µL of compound (200 µg/mL) or control solution. The final concentration of compounds in the reaction mixtures were 18.2, 9.1, 4.5, 2.3 µg/mL. Cedrol, a known PAF antagonist was used as a positive control 0.1% DMSO in saline was used as a control. The final concentration of DMSO in the reaction mixture was fixed at 0.1% to avoid interference with the receptor binding studies. The reaction mixture was incubated at room temperature for 1 h. The free and bound ligands were separated by a filtration technique using Whatman GF/C glass fiber filters. The radioactivity was measured by a scintillation counter. The difference between total amounts of bound ^3^H-PAF in the absence and the presence of excess unlabeled PAF is defined as specific binding of the radiolabeled ligand. The IC_50_ values of the compounds were obtained from at least three independent determinations. Percentage inhibition of the sample was obtained by the following equation: 


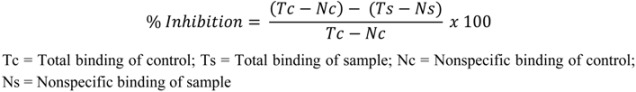


### 3.7. Statistical Analysis

All the data were analysed using the *Statistical Package for Social Sciences* (SPSS) software. Each sample was measured in triplicate and the data are presented as means ± standard deviation (SD). Probit programme was used to determine the IC_50_ values for the active pure compounds. The values were obtained from at least three determinations. Data were analysed using one way ANOVA. *P* < 0.05 was considered to be statistically significant.

## 4. Conclusions

Among the compounds isolated from the leaves of *Mitrella kentii*, chrysin showed a potent dose-dependent inhibitory activity on PGE_2_ production in human blood induced by LPS, indicating that it might directly inhibit COX-2 enzymatic activity. The results of this study indicate that the strong inhibitory activity of *M. kentii* on TXB_2_ production was due to polycarpol, acetylmelodorinol and stigmasterol which exhibited significant and concentration-dependent inhibitory effect on COX-1 activity. The strong PAF antagonistic effect of the plant was contributed by polycarpol and acetylmelodorinol which showed strong and dose-dependent inhibitory effects on PAF receptor binding. Acetylmelodorinol, chrysin and polycarpol have the potential to be used as lead structures for the development of potent anti-inflammatory agents. However, further studies need to be carried out to study their mechanisms of anti-inflammatory actions such as the cell free assays for COX enzyme activity and to find derivatives with maximum inhibitory effects. 
